# Spontaneous intracerebral haemorrhage associated with early-onset cerebral amyloid angiopathy and Alzheimer’s disease neuropathological changes five decades after cadaveric dura mater graft

**DOI:** 10.1186/s40478-023-01528-7

**Published:** 2023-02-24

**Authors:** Riccardo Milani, Lucio Aniello Mazzeo, Daniela Vismara, Ilaria Salemi, Emanuele Dainese, Emanuela Maderna, Elisa Pellencin, Marcella Catania, Nicole Campanella, Giuseppe Di Fede, Giorgio Giaccone, Andrea Salmaggi

**Affiliations:** 1 Fondazione IRCCS Istituto Neurologico Carlo Besta, Unit of Neurology 5 - Neuropathology, Milan, Italy; 2 Ospedale Alessandro Manzoni, ASST di Lecco, Lecco, Italy

**Keywords:** Cerebral amyloid angiopathy, Alzheimer’s disease, Iatrogenic, Neurosurgery

## Abstract

Cerebral amyloid angiopathy (CAA) is a small vessel disease, causing spontaneous intracerebral hemorrhage (ICH) in the elderly. It is strongly associated with Alzheimer disease (AD), as most CAA patients show deposition of Aβ—i.e. the basic component of parenchymal Alzheimer amyloid deposits—in the cerebral vessels. Iatrogenic early-onset CAA has been recently identified in patients with a history of traumatic brain injury or other cerebral as well as extra-cerebral lesions that led to neurosurgery or other medical procedures as intravascular embolization by cadaveric dura mater extracts many years before the first ICH event. In those patients, a transmission of Aβ seeds from neurosurgical instruments or from cadaveric dura mater exposure was suggested. We report a 51-year-old woman with unremarkable family history who presented abruptly with aphasia and right hemiparesis. A cerebral left lobar haemorrhagic stroke was documented by neuroimaging. Accurate anamnesis revealed a neurosurgical procedure with cadaveric dura mater graft at the age of 2 years for an arachnoid cyst. The neuropathological examination of the cerebral parietal biopsy showed severe amyloid angiopathy in many leptomeningeal and cortical vessels, as well as abundant parenchymal Aβ deposits, neurofibrillary tangles and neuropil threads. The mechanism involved in the human-to-human transmission of the Aβ proteinopathy remains to be clarified. In our patient the cadaver derived dura used for grafting is a very strong candidate as the source of the transmission. A systematic monitoring of individuals who have had neurosurgical procedures in early life, especially those involving cadaveric dural grafts, is required to determine the ratio of those affected by CAA many years later and unaffected. Moreover, our report confirms that in addition to vascular and parenchymal Aβ pathology, neurofibrillary changes indistinguishable from AD may develop in specific conditions with long latency period from the neurosurgical or embolization procedure.

## Introduction

Cerebral amyloid angiopathy (CAA) is characterized by misfolded amyloid Aβ deposits within blood vessels of the brain and leptomeninges. CAA is an important cause of lobar intracerebral haemorrhage (ICH) in the elderly [5]. Moreover, CAA is associated with ischemic cerebrovascular events, inflammatory leukoencephalopathy and cognitive impairment. The possibility of iatrogenic transmission of amyloid Aβ in humans by a prion-like mechanism has been initially described in iatrogenic Creutzfeldt–Jakob disease, and later in young adults with early onset CAA who had a history of neurosurgery or other invasive medical procedures in early life [2–4, 8–11, 13, 15]. After the first pathological description of presumed Aβ transmission in humans [11] and subsequent experimental confirmation [14], cases of iatrogenic CAA have been increasingly described. Herein, we report a case of early-onset CAA five decades after a cadaveric dura mater graft who underwent cerebral biopsy and showed the presence of neurofibrillary pathology beside Aβ deposition.

## Case presentation

A 51-year-old woman was admitted to the emergency department due to sudden loss of consciousness and left limbs weakness. Her previous medical history was unremarkable except for a neurosurgical procedure of arachnoid cyst’s evacuation and cadaveric dura mater grafting at the age of 2 years. At the time of this first acute neurological event, the patient had no sign of cognitive impairment. There was no family history of brain haemorrhage or neurodegenerative diseases.

**Fig. 1:**
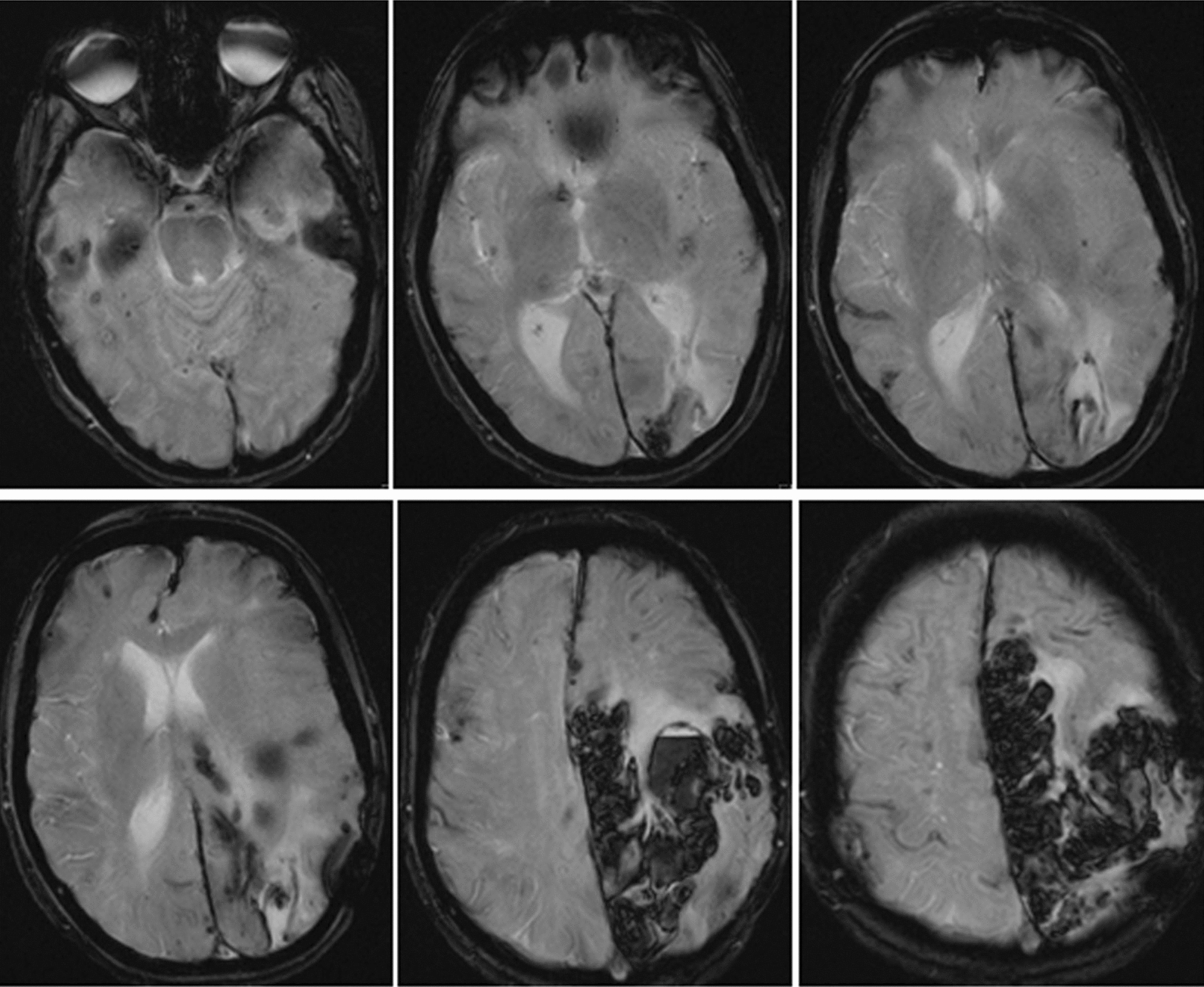
MRI T2* sequences showing a wide left fronto-temporo-parietal haematoma with several bilateral microbleeds

The neurological examination showed spared level of consciousness, expressive aphasia with preserved word comprehension, left homonymous hemianopia, left central facial palsy and left limbs weakness. A CT scan documented a wide intracerebral haematoma in the left frontal and temporal lobes with a 12 mm midline shift, and a malacic cavity in the left temporo-occipital lobe. The next day, a lowered level of consciousness developed, and left fronto-temporo-parietal osteodural decompression was performed; then, the patient was admitted to Intensive Care department. Brain MRI showed a lobar haemorrhage in the left fronto-temporal region, regardless of the arterial territory distributions, with several bilateral lobar microhaemorrhages on T2* sequences and diffuse T2 hyperintensity of the white matter, suggestive of ischemic leukoencephalopathy (Fig. 1). MRI angiography and conventional cerebral angiography did not show any vascular malformation. Diffusion tensor magnetic resonance imaging documented uncrossed pyramidal tracts (not shown) that could explain the occurrence of ipsilateral motor deficits.

The level of consciousness slightly improved over the following days, while persisted expressive aphasia and hemiplegia of the left limbs. Three months after the clinical onset, a sudden worsening of the responsiveness developed and a novel intracerebral haematoma in the left frontal lobe with a 2 cm subfalcine herniation was discovered on CT scan.

**Fig. 2:**
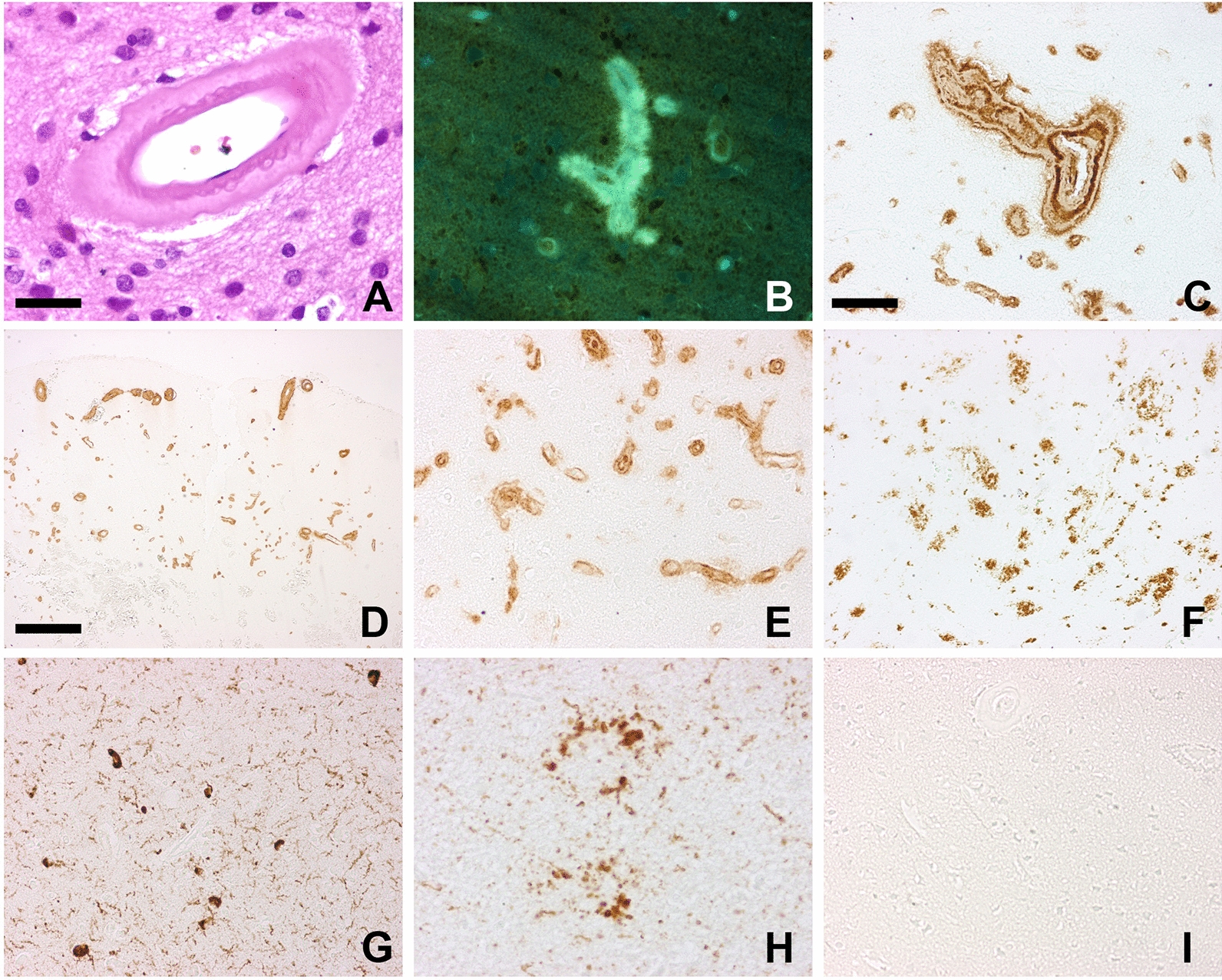
Neuropathologic findings of the cerebral biopsy. Severe amyloid angiopathy appeared as thickening of the wall of parenchymal arterioles (**A** Haematoxylin &Eosin) where amorphous material was present, exhibiting yellow/green fluorescence after thioflavine S treatment (**B**) and immunopositivity for Aβ (**C** 4G8 mouse monoclonal, 1:2000, after 80% formic acid for 20 min). The antibody specific for Aβ40 (mouse monoclonal, Covance, 1:1000, after 80% formic acid for 20 min) strongly decorated the amyloid-laden vessels (**D**), including capillaries (**E**). Aβ deposits were also abundant in the neuropil and these were intensely immunolabeled by anti-Aβ42 (**F**). Tau pathology was present, appearing as neurofibrillary tangles and neuropil threads (**G**) and clusters of dilated neurites (**H**) immunopositive for anti-phosphorylated tau antibody AT8 (mouse monoclonal, Biosource, 1:300). α-synuclein inclusions were absent (**I**). Immunolabeling was visualized by the Envision Plus/Horseradish Peroxidase System (DakoCytomation) using 3–3’-diaminobenzidine (brown reaction product) as chromogen. Bar in A = 35 µm (A, B and H are the same magnification); bar in C = 75 µm (C, E, F, G and I are the same magnification); bar in D = 350 µm

A cerebral biopsy from the left parietal lobe was performed, and the neuropathological examination revealed severe CAA in many leptomeningeal and cortical vessels, whose walls were laden by eosinophilic, amorphous material, yellow/green fluorescent after thioflavine S and immunoreactive for Aβ (Fig. 2A–C). The immunostaining with specific antibodies disclosed that Aβ40 was consistently represented in the vascular amyloid deposits (Fig. 2D), also in capillaries (Fig. 2E), while Aβ42 decorated the abundant parenchymal Aβ deposition (Fig. 2F). Neurofibrillary tangles, neuropil threads and tau-positive neurites around plaques were also present (Fig. 2G, H). Immunolabeling for α-synuclein (4D6 monoclonal antibody, 1:1000, Signet) (Fig. 2I) and Transactive Response DNA binding protein 43 (TDP43) (monoclonal antibody for phosphorylated TDP43, 1:1000, CosmoBio) was negative.

Neurological examination six months after the onset showed longstanding expressive aphasia and left limbs’ hemiplegia.

Genetic testing excluded known mutations of genes involved in hereditary Aβ-CAA (*APP, PSEN1; PSEN2*). The *APOE* genotype was ε2/ ε3.

## Discussion and conclusions

A recent review [3] listed 23 patients with highly suspected iatrogenic CAA, excluding two cases that were identified post-mortem without any clinical details. The median age of onset was 37.7 years, and 73.9% of the patients were male. The most common neurological manifestation was ICH (87.0%), recurrent ICH (65.2%), cognitive impairment (39.1%), seizures (26.1%).

Evidence of Aβ transmission in animal models had been demonstrated for many years [1, 6, 7], and the spread of Aβ proteopathic seeds through dural grafts and/or neurosurgical instruments could explain in most the cases the transmission of Aβ pathology between human subjects [12].

Alternative possibilities are disturbance of clearing system of cerebral Aβ, such as glymphatic system and intramural periarterial drainage pathways [8, 9]. but this is made unlikely by the description of cases of iatrogenic CAA after documented prior exposure to dura tissue that did not occur by neurosurgical grafting but by peripheral embolization of its extracts [2–4, 15].

Most of these reported iatrogenic cases were CAA [3], but a recent article showed that tau pathology similar to that of patients with Alzheimer disease can develop in patients with iatrogenic Aβ pathology after incubation period exceeding 3 decades [10].

Our report enlarges the number and the data available about patients with such a scenario. With a latency period between the neurosurgical procedure and the onset of the first neurological signs of 49 years, our patient represents the longest incubation period reported until now for iatrogenic Aβ cerebral amyloidosis.

The case reported here confirms that in addition to vascular and parenchymal Aβ pathology, neurofibrillary changes indistinguishable from AD may develop in specific conditions with long latency period from the neurosurgical or embolization procedure.

Therefore, history of neurosurgical and embolization procedures with the use of dura in young age and any other therapeutic procedures involving the use of potentially contaminated biological materials should be carefully searched for not only in patients who developed early-onset CAA but also Alzheimer disease.

## Data Availability

The datasets during and/or analysed during the current study available from the corresponding author on reasonable request.
